# Minimum entropy collaborative groupings: A tool for an automatic heterogeneous learning group formation

**DOI:** 10.1371/journal.pone.0280604

**Published:** 2023-03-15

**Authors:** Toni Vallès-Català, Ramon Palau

**Affiliations:** 1 Centre d’Estudis Superiors de l’Aviació (CESDA), Reus, Catalonia, Spain; 2 ARGET Research Group, Faculty of Education Sciences and Psychology, Universitat Rovira i Virgili, Tarragona, Catalonia, Spain; Rose-Hulman Institute of Technology / Indiana University, UNITED STATES

## Abstract

For some decades now, theories on learning methodologies have advocated collaborative learning due to its good results in terms of effectiveness and learning types and its promotion of educational and social values. This means that teachers need to be able to apply different criteria when forming heterogeneous groups of students and to use automated techniques to assist them. In this study, we have created an approach based on complex network theory to design an algorithm called Minimum Entropy Collaborative Groupings (MECG) in order to form these heterogeneous groups more effectively. The algorithm was tested firstly under a synthetic framework and secondly in a real situation. In the first case, we generated 30 synthetic classrooms of different sizes and compared our approach with a genetic algorithm and a random grouping. In the latter case, the approach was tested on a group of 200 students on two subjects of a master’s degree in teacher training. For each subject there were 4 large groups of 50 students each, in which collaborative groups of 4 students were created. Two of these large groups were used as random groups, another group used the CHAEA test and the fourth group used the LML test. The results showed that the groups created with MECG were more effective, had less uncertainty and were more interrelated and mature. It was observed that the randomized groups did not obtain significantly better LML results and that this cannot be related to any emotional or motivational effect because the students performed the test as a placebo measure. In terms of learning styles, the results were significantly better with LML than with CHAEA, whereas no significant difference was observed in the randomized groups.

## Introduction

In recent decades, interest in cooperative and collaborative learning has extended from primary schools up to higher education institutions, and from physical to online education. Cooperative learning is based on the ideas of Dewey [1], who believed that learning has a social component and that interaction between students results in better learning, ideas that are supported by the systemic theory of Vygotsky [2] on how learning improves through engagement with other students. From Dewey, in the first half of the 20th century, it began to be demonstrated that group learning was better than individual learning [3].

This marked the beginning of research into group learning and the relationships that are established, which continued throughout the 20th century up to the findings of Johnson and Slavin [4, 5], who improved theories on social interdependence and cognitive, socio-cognitive and behavioral development, and then on to current studies [6–8], who demonstrated the general effectiveness of cooperative learning in schools and higher education.

One of the challenges when institutions and educators want to apply these methodologies is the procedure used to create the student groups. Studies show that team self-selection is not effective, and that groups must be formed by the teacher and expert for teams to be effective [9]. There are different methods and criteria for forming groups that are not based on self-selection. Some authors focus on individual students’ characteristics such as ability level [10], demographic data [10, 11], roles [11, 12] or learning styles [13–15], whereas others focus on group characteristics such as motivation and group stability [16] or interactive behaviors [17]. In terms of types of groups, Vygotsky was the first of many to demonstrate the benefits of heterogeneous groups for students of high or low ability [2]. According to Dascalu et al. [18] there are 3 main ways of forming groups. The first two, namely self-formed groups and random groups made by the teacher, can be said to occur without the application of any specific method or criteria. The third way is to apply criteria through algorithms. These criteria can be based on academic results, profile, cognitive level, motivation, social preferences, roles in the group and goals [19]. Two ways of creating groups can be identified in this manner; the first involved creating groups based on the goals of the individual members and the second was based on determining first the group goals. Certainly, further research into group formation is required over the coming decade [20, 21].

One of the criteria used is learning styles [13–15], which refers to categorizing learners into one or more styles in order to adapt teaching and learning strategies and thus improve results [22]. Honey and Mumford, based on the work of Kolb, identified four learning styles, namely the Activist, the Theorist, the Pragmatist and the Reflector [23, 24]. Although these have been widely recognized among teachers, doubt has recently been cast on their scientific validity by several studies [22, 25, 26], all of whom assert that there is little empirical evidence to support the theory [27, 28]. In contrast, Johnston CA understood the learning process as a practical function of the brain-mind connection and metacognition that helps teachers and learners improve learning success [29]. This model of learning based on the tripartite theory of the mind (cognition, connation and affect) attributes specific behaviors to their internal interaction within each of the four discrete operational processes of learning patterns, named Sequence, Accuracy, Technical Reasoning and Confluence [30]. To identify them, the same author developed an instrument with forced-choice (Likert) and open-ended responses that enabled a qualitative and quantitative analysis of a student’s learning pattern. This tool has evolved into different versions for different educational levels [31].

Continuing with the grouping methods, when there is a large number of students (higher education groups), putting them into groups on the basis of more than one criterion seems to be too complex to be solved by humans [10, 32, 33], and this is where computers and algorithms come into the picture. Several algorithms have been developed to automatically group students heterogeneously, but a predominant approach cannot be found. Instead, there is a wide variety in the nature of the algorithms and the attributes used to form heterogeneous groups. To name a few: Ounnas et al. [11] use a logic programming to assess the group formation problem as a constraint satisfaction problem, differentiating students with different ontologies they define; Balmaceda et al. [34] differentiate between hard and soft constraints, taking into account psychological styles; Erkens et al. [35] differentiate students with their writing style, using a text mining approach; and Konert et al. [36] use the GroupAL algorithm to group students based on a distance measure with a set of weighted criterion.

Notwithstanding, there is a tendency in the literature to use mainly genetic algorithms [10, 15, 32, 37–41] and also particle swarm optimization algorithms [42–45]. In a genetic algorithm, the partition of the groups evolves by swapping the students in the different groups in a three-step iteration—selection, crossover and mutation—to achieve an optimal grouping of students. The idea behind the particle swarm optimization is to model the social behavior of students as fish schooling or birds flocking collaborative working to solve a complex problem, find a food source or scheduling problems. In a review by Maqtary et al. [20] 60% of the 30 studies considered were based on genetic algorithms, but neither of them is an ideal process in Computer Supported Collaborative Learning (CSCL), and further approaches are needed for an improvement [20].

There is also another starting point that is not commonly used: one based on complex networks and which is the approach that we will present here.

### The use of complex networks in automatic group formation problem

Complex networks science reveals information from a complex system by considering it instead of considering its elements separately. For instance, we can predict how a disease spreads via the global air transportation network [46] or predict individual preferences for online products with recommender systems [47]. A network consists only of the definition of its elements (nodes) and the connections between them (edges). This flexibility enables networks to be applicable in a wide range of fields, meaning that there are, among others, transportation networks, metabolic networks, neural networks, peer-to-peer networks, and social networks [48]. In the present study, we will define a social network consisting of students and define a metric to connect them according to their similarities.

The problem of automatic group formation has been assessed using complex networks in the past. Chen and Kuo [37] get the clustering coefficient from social networks to compare their non-network based approach; Bekele and McPherson [49] use Bayesian networks where nodes are concepts instead of students, to predict group performance; Balmaceda et al. [34] take into account three students’ features: psychological styles, team roles and social networks; and Wi et al. [41] build a social network of members based on co-authorship, to form groups with a genetic algorithm. However, those studies mainly use complex network science to obtain a measure that quantifies the quality of the groups and not for group formation in itself.

Some approaches use network flow theory to find an optimal solution and form student groups. Graf and Bekele [42] applies an ant colony optimization from the family of particle swarm optimization methods to directed networks to obtain the groups, and Bhadury et al. [50] uses the classic dining philosopher’s problem to obtain student groups with a bipartite network, where nodes can be either students or groups. However, the computation of group quality in these studies is the same as that used for group formation, hindering the comparison with other approaches.

Finally, there are few studies that use networks to form the groups and a different quality group measure. Alberola et al. [51] used machine learning to build a student network with Belbin roles, and then performed several iterations of the group formation getting feedback after each iteration. Also, Srba and Bielikova [17] proposed automatic group formation in several dynamic groups iterated using a clustering algorithm on a matrix (similar to the adjacency matrix of a network). These approaches need several iterations to be used, so the main disadvantage is that the teacher is forced to plan different group activities to use them.

Then, a necessity arises of a complex network-based approach to automatically build collaborative learning groups, that can be comparable to other approaches and can be used in a single group activity.

### Objectives and hypotheses

The present study aims to create and develop an automatic heterogeneous group creation tool based on complex networks and entropy measurements, and to evaluate its performance for creating heterogeneous groups in cooperative learning.

More specifically, in this study the hypotheses are:

H1: The automatically created groups will obtain better learning performances than the randomized groups.
  - H1a: the automatically created groups based on CHAEA obtain better learning performances than the randomized groups.
  - H1b: the automatically created groups based on LML obtain better learning performances than the randomized groups.

## Materials and methods

In this section, we explain how we built an algorithm in order to partition the students on the basis of the following items: (i) a list of N student’s names; (ii) numerical information of the attributes of each student (provided by a questionnaire); (iii) the number of students per group (ng).

### Building a student network

**Fig 1:**
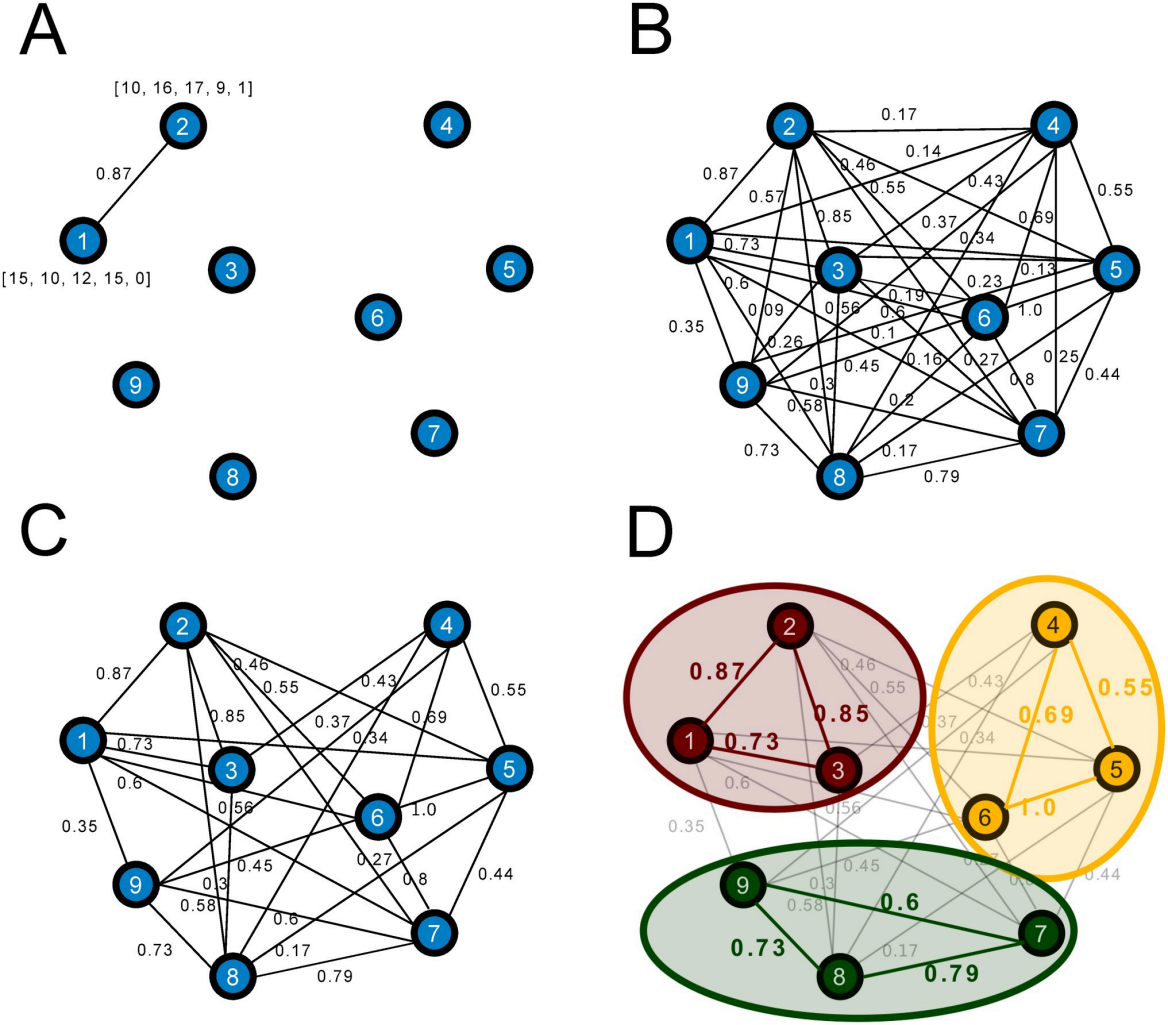
How we build a weighted network of a classroom. Students are represented as nodes in a network. In this example assume 9 students and 3-member groups. **A**: The first step is to compute the dissimilarity between a pair of students given their attribute vectors. As an example, student 1 and 2 answered the same questionnaire obtaining *(15*, *10*, *12*, *15*, *0)* and *(10*, *16*, *17*, *9*, *1)* respectively, 4 values for defining the learning style and the last one for gender. Then the dissimilarity between student 1 and 2 is computed with Eq (1). **B**: Complete graph after applying the previous procedure for all pairs of students in the classroom to obtain all dissimilarities in the network. **C**: Reduced network, we remove one third of the total edges (those with lower dissimilarity) to reduce computational cost. **D**: Resultant partition after fitting several Stochastic Block Models and finding the optimal one with minimum entropy, note that when nodes in the same group have higher values of dissimilarity, then heterogeneous groups are formed.

Let *{n*_*1*_, *n*_*2*_,…, *n*_*N*_*}* be the set of _N_ nodes of a network of students, each node representing a student in a classroom of N students. Let *{e*_*11*_, *e*_*12*_… *e*_*ij*_,… *e*_*NN*_*}* be the set of edges of our network, edge eij being the dissimilarity between student i and student j. This set of edges can be inserted into a matrix called the adjacency matrix *A* of the network, with the value *A*_*ij*_ of the adjacency matrix being the value of *e*_*ij*_ (for an example, see Fig 1A where the dissimilarity between students 1 and 2 is *e*_*12*_ *= A*_*12*_ *= 0*.*87*).

To compute the dissimilarity between two students, we use the information from the questionnaire answered by each student. We use two different questionnaires: CHAEA and LML, introducing an extra question for gender. We choose those questionnaires because they have been used in other studies to determine different attributes of students, for instance Cela-Ranilla et al. [52] and Thania et al. [53].

The Honey-Alonso (CHAEA) questionnaire [54], consists of 80 binary questions that are split into 4 different learning styles. For example, a student can have 16 activist points, 11 reflector points, 13 theorist points, and 17 pragmatist points. We can represent this information for one student with a 4-dimensional space vector containing 4 attributes *(15*, *10*, *12*, *15)*; another student may then have a different learning style configuration represented with another vector, for example *(10*, *16*, *17*, *9)*. By adding an extra question for gender, a 5th attribute, we obtain a 5-dimensional space vector for each student. In our example, therefore, if the first student is a female the attributes are *(15*, *10*, *12*, *15*, *0)*, and if the second student is male, they will be *(10*, *16*, *17*, *9*, *1)*.

The Let Me Learn (LML) [30] questionnaire consists of 28 questions that can be answered in a 5-point Likert scale, answers are split into 4 learning patterns (sequential, precise, technical reasoning and confluent). Note that we can represent this information again in a 5-dimensional vector space (4 patterns and gender).

To compute the dissimilarity between two students we compute the difference between two vectors, and we compute a normalized weighted squared sum to obtain a unique value from the vector difference. Then to compute the dissimilarity between students i and j we use the following equation:

$$e_{ij}=\sqrt{\frac{\sum_{k=1}^{l}w_{k}\;\cdot \;{\left(i_{k}-j_{k}\right)}^{2}}{max_{n,m\in N\times N}\left\{\sum_{k=1}^{l}w_{k}\;\cdot \;{\left(n_{k}-m_{k}\right)}^{2}\right\}}}$$ (1)

Where we have the information of *l* attributes for each student, with *l* different weights *w*_*k*_ *(k = 1*,*…*,*l* one for each attribute). Konert et al. suggests that algorithmic formation of learning groups should allow criteria weighting [36], thus we permit the user to specify weights. Observe that the difference between the attributes is squared to avoid negative values. Finally, note that the value is normalized by the maximum dissimilarity among all possible pairs of students *n*, *m*.

In the latter example, we obtain a vector difference of *(5*, *-6*, *-5*, *6*, *-1)*. Note that gender is underrepresented since the squared sum of the vector difference is 123 and the gender difference only accounts for 1. Therefore, a higher weight is needed for gender: we use the mean of the 4-dimensional vector space as a weight for gender, to avoid underrepresentation of this variable. In our example, the mean of both *(15*, *10*, *12*, *15)* and *(10*, *16*, *17*, *9)* is 13, when using it as the weight for gender we obtain a dissimilarity of 0.87 (assuming that the maximum dissimilarity in our example is 178).

This example is illustrated in Fig 1A; to build the whole network we will compute the dissimilarity between all pairs of students within a classroom, shown in Fig 1B. The purpose of this study is to provide heterogeneous groups formed by members that have different attributes (for instance, different learning styles/patterns). We are thus interested in grouping together those students that have high values of dissimilarity.

### Weighted stochastic block models

There are several approaches to partitioning the nodes into densely connected groups [55], i.e., groups that have more connection between members of the group than connections with members of other groups. One of those approaches is the family of Stochastic Block Models (SBM) [56], an approach from social science in which the groups of nodes are called Blocks, and Blocks group together those nodes that have similar patterns of connection. When you have all nodes in different Blocks, you obtain a partition of the network that is called a Model. Different Models of the network are different ways of distributing nodes into groups. Finally, they are called Stochastic because the probability of connection between each pair of nodes depends only on the probability of connection of the Block they belong to.

In this study, we need the variant for weighted networks. In simple networks, each pair of nodes are either connected or not connected in a binary relation; conversely, in a weighted network we can assign values to the edges of the network. In our case we assign the dissimilarity of two students as the weight of the edges (see Fig 1B).

Since we can compute the dissimilarity of all pairs of students, then all the edges have non-zero value and we obtain a network that is a complete graph. To reduce the computation cost of a complete weighted graph, we remove one third of the total number of edges, those with lowest dissimilarity (see Fig 1C). From this weighted network we can propose different Models (different partitions of students into groups), Fig 1D shows an example of a Model, in fact it is the optimal one. In the next section we explain how we reach it.

### Minimum entropy collaborative groupings

We have defined a network of dissimilarities that represent a classroom and describe the relations between the students. We have also defined an SBM that is a way of splitting the students into different groups. Since there is a wide variety of possible SBMs that we can use (i.e., students in a classroom can be grouped in several ways), we need a measure in order to determine how heterogeneous the groups are, and we propose the entropy measure. Entropy is a measure that comes from the field of thermodynamics, it can be used to measure the degree of disorder that a state may have in a system of atoms or molecules. In network science, entropy has been used before with SBMs [57]: the system consists of nodes instead of atoms (in our case students), and one state will be one specific partition of students into groups. A low entropy state will represent groups of students with a low degree of disorder and therefore groups of students that have similar patterns of connection. It might seem that we would obtain homogeneous groups because we group together students with similar patterns of connection; however, our network is built with dissimilarity edges, meaning that the students that we group together are those with more dissimilar connections, thus forming heterogeneous groups.

Our approach is called Minimum Entropy Collaborative Grouping (MECG) because we obtain collaborative groups by minimizing the entropy of the network system. Since we are dealing with a weighted network, we use the approach by Peixoto [58] to compute the entropy of a weighted SBM (see S1 Appendix for further information). We then use simulated annealing to find the optimal SBM with minimum entropy. We show the steps that our algorithm follows given three inputs: the list of *N* students with questionnaire answers, the number of attributes, and the number of members we want per group (*ng*).

1. Read a list of students containing numerical information about CHAEA or LML learning styles/patterns and gender.
2. Build dissimilarity weighted network.
3. Repeat 100 iterations:
  - Propose an initial random partition of the students with *ng* specified.
  - Compute entropy of this SBM partition.
  - Repeat *N* iterations:
    - Switch 2 students from different groups.
    - Compute entropy *S*_1_ of the SBM partition candidate, keeping the previous partition with entropy *S*_0_.
    - Accept the change if *S*_1_ *< S*_0_ or with a random probability proportional to $e^{-(S_{1}-S_{0})}$.
    - Check local minimum entropy with global minimum entropy, saving new group partition if new global minimum entropy has been found.
4. Show global minimum entropy group partition.

The code is available online [59].

### Measuring performance

To measure the performance of a group, we are interested in how well a group can succeed at the task set, meaning that we need to measure the group’s effectivity.

Additionally, there are different dimensions to consider when measuring how well different people form a group. We use the 6 scale questionnaire by Navarro et al. [60] consisting of 56 questions from a 5-point Likert-scale that evaluate effectiveness, entitativity, uncertainty, interrelationship, maturity and potency. Entitativity refers to the degree to which a group is seen as an entity by its members. Uncertainty is high when the task goal is not connected with the result achieved, more clarity in this connection will mean less uncertainty (note that low values in this measure represent better performance for a group). Interrelationship refers to the degree to which the members establish interpersonal feelings and behaviors with each other. Maturity situates the group on a continuum from non-collectivism to collectivism, in high maturity groups there is more interaction between the members. Potency refers to the degree of confidence in the group to succeed at the task goal [61, 62].

### Experiments

Two different experiments are conducted: first we apply the approach in a non-real case, with synthetic generated classrooms, where several students’ learning styles are randomly generated; then we apply the approach in real classrooms.

#### Synthetic classrooms

We generate 15 random classrooms of different sizes (5 of 20 students, 5 of 30 students and 5 of 50 students), in which students have been generated following a normal distribution, given the mean and standard deviation of learning styles/patterns of both the questionnaire CHAEA [54] (Active: 10 ± 3.25, Reflexive: 14.5 ± 2.87, Theorical: 11.9 ± 3.16, Pragmatic: 12.9 ± 3.12) and the questionnaire LML [30] (Sequential: 26.3 ± 4.1, Precise: 25 ± 3.4, Technical: 21.4 ± 4.7, Confluent: 22.0 ± 3.1).

For each generated classroom, we search for the optimal partition of students into groups using our approach MECG; and, for each size, we compute the mean of the entropies of each optimal partition of the 5 classrooms. We consider as size groups the 3-member groups and 4-member groups, separately.

Analogously, we repeat the process with two other approaches for comparison: (i) grouping the students randomly; (ii) the genetic algorithm. The former is computationally simple, but for the latter we elaborated an algorithm based on the work by Moreno et al. [39]. We selected the genetic algorithm because it is severally used in the literature for automatic group formation [10, 15, 20, 32, 37–41].

#### Experiment with real students

**Fig 2:**
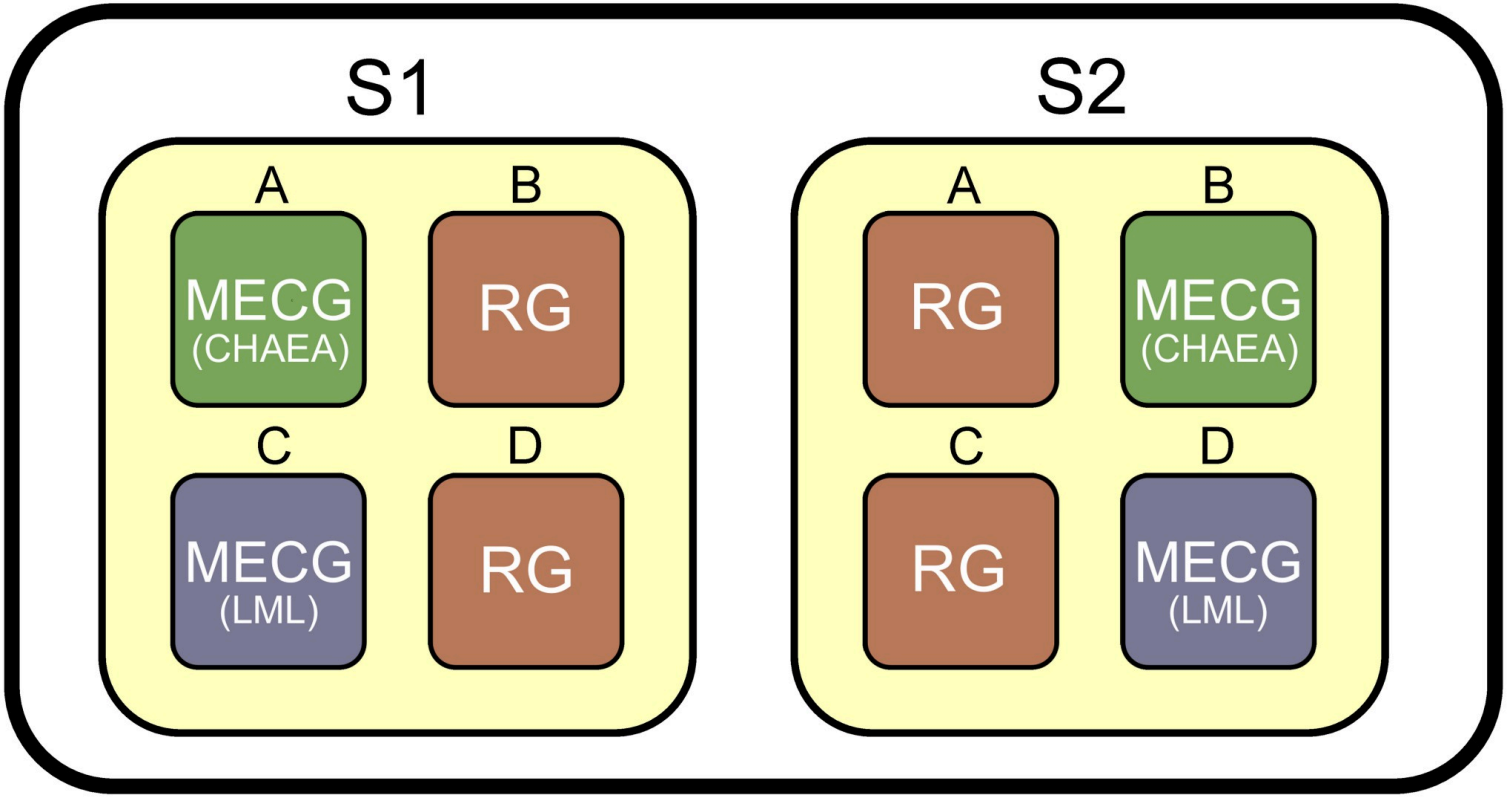
Experiment scheme. Students are divided into 4 different classrooms (A, B, C, D) within each subject (S1, S2). The same student is grouped randomly in one subject, and then grouped with our approach in the other subject (without their knowledge). Classrooms that are grouped randomly are shown in red (RG), and classrooms that are grouped with Minimum Entropy Collaborative Groupings (MECG) are shown in green (CHAEA) or blue (LML).

We tested our approach on a real case of 200 students (mean age of 32 ± 8 years) studying two specific subjects from a master’s degree in teacher training. For both subjects, students must present several teamwork projects; all the groups remain fixed throughout the academic year. The students first answered both the CHAEA and the LML questionnaires. Then with this information we used our approach to split the students into 4-member group; some were grouped with MECG and others were randomly grouped as a control group (RG) (see Fig 2). We used two different subjects from the master’s degree (subject S1 and subject S2), each of which consist in 4 classrooms of approximately 50 students (classrooms A, B, C and D). In general, the same students conform the same classrooms in different subjects, thus we could group class A for S1 using our MECG approach, and then randomly group the same students in class A for subject S2. This meant that in total we had 8 classrooms that were grouped, as shown in Fig 2. All participants agreed to participate by clicking the online version of the CHAEA and LML questionnaires described above. The study is certified to be carried out following the principles and evaluation criteria of the ethics committee CEIPSA, from Universitat Rovira i Virgili, with code CEIPSA-2022-PR-0018.

## Results

The results are shown in the following two sections:

### Synthetic classrooms

**Fig 3:**
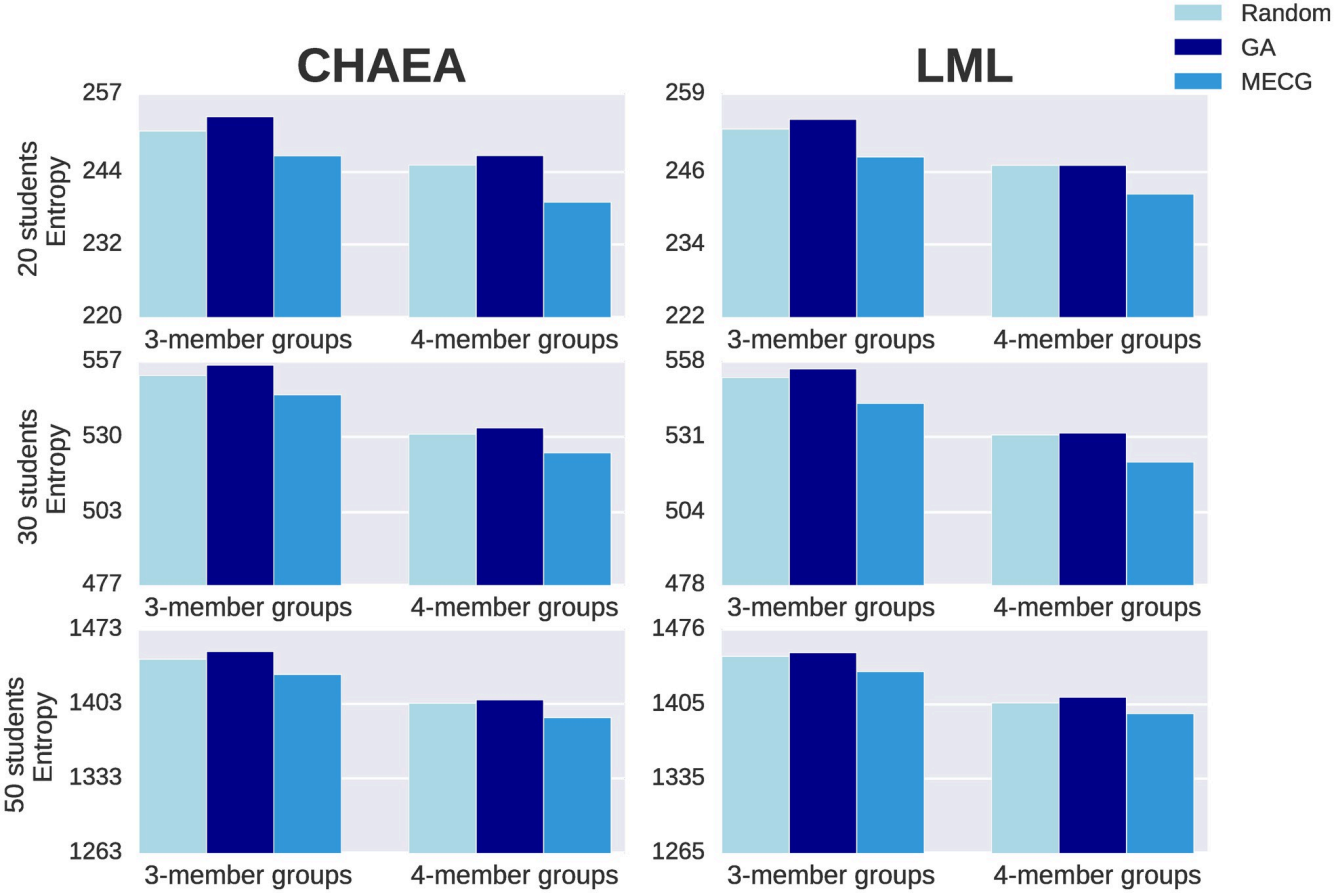
MECG partitions have lower entropy. We generate 15 random classrooms of different sizes (5 of 20 students, 5 of 30 students and 5 of 50 students), in which students have been generated following a normal distribution, given the mean and standard deviation of learning styles/patterns of both CHAEA [54] and LML [30] questionnaires. For each generated classroom, we search for the optimal partition of students both into 3-member groups and 4-member groups, separately, using our approach to form Minimum Entropy Collaborative Groupings (MECG); and, for each size, we compute the mean of the entropies of each optimal partition of the 5 classrooms (The standard deviation is not shown because it is too low to be appreciated). Analogously, we compute the mean entropy for each size with two other approaches to compare with: (i) grouping the students randomly; (ii) a genetic algorithm that we elaborated based on the work by Moreno et al. [39]. Observe that, in all the cases (different classroom size, 3-member or 4-member groups, CHAEA or LML), the entropy of our approach MECG is lower.

Fig 3 shows that, in all the cases (different classroom size, 3-member or 4-member groups, CHAEA or LML), the entropy of our approach MECG is lower. Thus, the MECG technique groups together students that are dissimilar (given a dissimilarity adjacency matrix).

### Experiment with real students

When students answered the questionnaire by Navarro et al. [60], the Cronbach Alpha for Subject 1 was 0.87, and for Subject 2 was 0.93. Table 1 shows the results obtained by comparing the answers given for the 6 scales of the questionnaire by the MECG and the random grouping. In general, studies assessing the group formation problem that involve real students use as a control group a random grouping [17, 35, 39, 40] or also self-selection [14, 37–40]. The dataset is available online [63].

**Table 1:** Performance of Minimum Entropy Collaborative Groupings (CHAEA or LML) compared to Random groups (RG).

Comparison	Effectiveness	Entitativity	Uncertainty	Interrelationship	Maturity	Potency
CHAEA vs RG	1.32(0.219)	0.885 (0.377)	-1.65 (0.0994)	-0.377 (0.706)	**2.16 (0.0309)**	0.763 (0.446)
LML vs RG	**4.32 (1.72·10** ^**−5**^ **)**	1.66 (0.0983)	**-2.87 (0.0042)**	**2.46 (0.0141)**	**3.16 (0.0017)**	1.73 (0.0837)

Table 1 shows the t-t paired test to compare the performance when the same student was grouped randomly versus an MECG partition (CHAEA or LML). Performance is observed from 6 scale: Effectiveness, Entitativity, Uncertainty, Interrelationship, Maturity and Potency. The statistic is shown in bold when the p-value (shown in parenthesis) is lower than 0.05. Observe that LML heterogeneous groups perform significantly better in 4 out of 6 scales, therefore heterogeneous groups are more effective, have less uncertainty, are more interrelated and mature (note that low uncertainty means better connection between the task goal and the result achieved). Moreover, groups formed randomly do not perform significantly better in any of the scales than LML heterogeneous groups. On the other hand, observe also that CHAEA heterogeneous groups perform significantly better only in maturity. In general, their performance is indistinguishable compared to a random grouping, at least in this study.

The hypothesis H1: the automatic groups obtain better learning performances than the randomized groups is confirmed, as is hypothesis H1b, the automatic groups based in LML obtain better learning performances than the randomized groups.

## Discussion

The first aim of this work was to create and develop an automatic heterogeneous group creation tool based on complex networks. We called the resulting tool MECG (Minimum Entropy Collaborative Groupings). In doing so, we seem to be addressing a real need regarding the most effective way to divide up big groups heterogeneously. One of the reasons for this, as Hwang et al. [32] described, is that this is too complex, especially when more than one criterion is used. Therefore, the development of such a tool would be beneficial for students as it increases their motivation and reduces dropout rates, thus making it easier for teachers to work [33].

Konert et al. [36] suggests that algorithmic formation of learning groups should include calculations of group quality, and Cen et al. [8] highlights the need for quantitative rigor to prove the benefits of collaborative learning. Therefore, the second aim of this study was to evaluate the performance of the MECG with a group of 200 teacher training master’s students in two subjects and thus compare learning styles/patterns of collaborative groups with randomized groups. Students did the test as a placebo measure to avoid emotional or motivational effects. For performance, we measured 6 scales with the questionnaire from Navarro et al. [60].

Forming groups in a non-randomized way must consider the relationship between different students in a classroom. Complex networks are appropriate to solve this kind of problem, with which we can define the relationship between the different students. Then the choice of this relationship determines which kind of groups we would like to obtain. In our approach, we focus on learning styles/patterns (using CHAEA/LML questionnaires) plus gender, but our approach is flexible enough to permit the use of any questionnaire. In this way, the constraints (or attributes) are not closed to a specific choice as is the case in previous attempts at automatic group formation that have used, for instance, fixed ontologies [11, 19], or fixed constraints and steps to follow [33]. The flexibility of networks was advantageous when evaluating computer-supported collaborative learning techniques [64].

There is a tendency to use distance computational methods such as genetic algorithms to assess the group formation problem [10, 15, 32, 37–41], while network-based approaches are rarely used for that purpose: in two recent reviews on group formation problem, Maqtary et al. [20] and Odo et al. [65] that have reviewed 30 and 48 studies respectively, only one approach based on networks is found [51]. In comparison with our network based approach, genetic algorithms also allow the attributes to be chosen by the user, but they often require determining parameters [39], or need a refinement for a binary variable such as gender [10]. Our approach is more flexible, no refinement or parameters are needed (excluding the questionnaire and weights chosen by the user), and extra attributes can be added without modifying the algorithm. Notwithstanding this, although the approach can handle an unlimited number of attributes, too many will diminish the effect of each attribute on the groupings. Thus, we recommend using no more than 7 attributes; this is in line with Ounnas et al. [11], who compared different computerized group formation approaches and recommended a maximum of 7 constraints. Moreover, compared to other clustering methods and tools, MECG is adaptable to different educational ages because it is independent of the initial questionnaires. It could be used with questionnaires designed for that age group if one wanted to use it in primary education, for instance. At the same time we can say that MECG can never be outdated, because when new versions of the questionnaires or better ones are detected, a quick and easy adaptation can be made.

Regarding the experiment, we provide a robust framework when including the same students in both the control and the experimental groups (in different classrooms), thus we can compare the performance of the groups with the same student in a paired t-test and reduce external factors. In contrast, most formation group approaches that were validated involving real students, have built the control and experimental groups with different students [10, 14, 35, 38–40].

Our approach to automatic group formation is not the first one related to complex networks, but as far as we are concerned, our study is the first to use entropy measure for this purpose. Moreover, our approach differs from others that only use complex networks science to obtain a measure of the quality of the groups [37, 41, 49], conversely, we use network science to build the groups and then for fairness we measure the quality of the group with a different tool (the 6 scales team performance). Finally, there are indeed few approaches using complex networks to build the groups: (i) Shih et al. [40] use the genetic algorithm method to form the groups, but introducing social network data in the crossover step; (ii) iterative groupings by Alberola et al. [51] or Srba and Bielikova [17], these approaches are appropriate for subjects with several group activities, since the groupings improve with the iterations of the algorithm, but are disadvantageous in subjects with few group activities because when there are fewer iterations the random groupings are closer. Our approach provides the optimal partition (concerning entropy) in one grouping, without the need of different group activities to iterate. In case several group activities are planned, the information of a previous grouping could be helpful for the next one [17, 51]. Our approach can also be used iteratively if this information is included in the input information of our algorithm to provide a different grouping in the next group activity. Another option is that our approach complements Alberola et al. [51] as an initial iteration.

We have shown that MECG provides students groupings with lower entropy with respect to those provided by a random grouping or a genetic algorithm, and it was shown in several situations (different classroom sizes, 3-member or 4-member groups, synthetic students generated given CHAEA or LML questionnaire usual answers). It is reasonable that our approach provides lower entropies since it was built for that purpose. We wondered what would happen if we computed the fitness value defined in Moreno et al. [39] for all the optimal partitions (the ones provided by the genetic algorithm, by our MECG, and also random partitions), results are shown in S1 Fig. We observed that our approach provides fitness values that are much higher than the genetic algorithm and the random partitions. This fitness value represents the inter-homogeneity between groups, so these results suggests then genetic algorithms provide more inter-homogeneous groups (see S1 Fig) but we suggest that MECG provides more intra-heterogeneous groups (see Fig 3), implying that when you minimize one variable the other is affected. Whether which is a better option cannot be decided with the fitness value nor entropy measure alone, a unified criterion should be used. Since there are no established criteria in the literature, we propose to compare the opinion of the team members with the 6 scale questionnaire by Navarro et al. [60]. This interesting future work will have to be assessed in real experiments instead of a synthetic framework.

As a first step, in this work we present a first comparison in a real situation between our approach and a random grouping. Results in Table 1 support that, at least using the LML questionnaire, our approach provides groups that work better than grouped randomly, suggesting that lower entropy groupings provide better performance groups.

### Conclusions

A principal conclusion is that the groups created with MECG are more effective, have less uncertainty, and are more interrelated and mature. Concerning the two questionnaires used, the results were significantly better with LML based on learning patterns [29] than with CHAEA based on learning styles [54], where we could not observe any significant difference with respect to the randomized groups. This may be explained with the conclusions of certain authors [22, 25, 26], who showed concerns about this theory and criticized the poor scientific evidence and validity of learning styles as a theory [22, 27, 28]. As this article has shown, the use of MECG in comparison to non-criterion grouping improves students’ learning. Therefore, when teachers use active learning methodologies where groupings are involved, a scientific evidence approach, as the one proposed here, should be used rather than a non-criterion one or a grouping based on a teacher’s subjective decision.

### Limitations

Some minor concerns have to be taken into account regarding the experiment with real students. The experiment was held during the 2019–2020 academic year, when the pandemic forced all the groups to work through online meetings. We achieved 88% participation, the other 12% were grouped randomly and not considered for the study.

In general, the groups consisted of 4 members, but in some classrooms a 5-member group or a 3-member group was required. Moreover, classrooms in different subjects were mainly formed by the same students, but some exceptions had to be considered: some students only attended one subject, and some students were in different classrooms depending on the subject. Nevertheless, around 86% of the students were in the same class in S1 and S2 and our results are based on this sample.

We compared our approach with a random grouping and a genetic algorithm in the synthetic experiment, while we could only compare our approach with a random grouping in the real experiment, as is usually done in the literature. In this study we could not include a genetic algorithm in the real experiment, where we would need the same student volunteers in three different subjects, simultaneously, increasing the complexity of the experiment.

### Future research

Our results suggest the usefulness of further studies applied to other educational levels such as secondary or primary education; although the groups at such levels are not 3usually that large, the demand and trend for group activities seems to be growing continuously. Another line would be to analyze which questionnaire and variables perform better. Since our flexible approach can adapt to any questionnaire, it would be possible to consider the Belbin roles used by other studies [11, 12, 51], or the Big Five test [10, 33]. Variables studied in previous studies could also be taken into account, for example schedule availability [33], previous grades, classmates’ preferences, etc. Also, the use of the gender variable to group students has to be studied; Ounnas et al. [11] suggest that a female should not be grouped alone with male members to avoid being stranded.

Additionally, on the one hand Konert et al. [36] suggest flexibility between homogeneous and heterogeneous groups, on the other hand Cen et al. [8] advocates for heterogeneous groups to favor collaborative learning. In a future work, Stochastic Block Models could be adapted to mixed heterogeneous groups to compare both attitudes. Finally, we have found a huge spectrum of group formation approaches, but they are compared with random or self-selection only [14, 17, 35, 37–40]. There is therefore a need to compare different approaches, with a group quality measure that should be different from the one used for group formation.

### Implications

Nowadays, there is increasing interest in the education sector in applying active methodologies that require collaborative work. The way groups are formed has historically been one of the weak points or points to improve, due to the fact that they have been made in a randomly on the one hand, or by affinity or friendship between the students or by the subjective criteria of the teacher. We therefore firmly believe that the contribution and implications of this article for the education sector can be:

For teachers: grouping is a crucial element of new learning methodologies based on active learning that needs to be done correctly. The task is too complex or even impossible to perform manually or without a specific tool. Therefore, the algorithm developed in this research brings to the table a new way of being able to group learners in a fast and scientifically based way. Teachers only must select the attributes they want to take into account for a heterogeneous grouping, in this study we considered CHAEA or LML questionnaires, but our approach is flexible enough to permit other questionnaires or even other variables such as students marks or time availability. After gathering this information from the students, the teacher can use our code [59] to automatically group the nodes, detailed information on how to use it is included in [63].

For researchers: grouping is not a new topic in research but not completely solved. This article will provide another point of view on how to do it and a starting point for further research in other contexts and especially in other educational stages such as primary and secondary education, where there is a lot of interest in collaborative learning.

For those interested: at a time of post pandemic, where they are questioning what kind of learning, what and how students can learn better from the experiences lived with confinements and forced online learning, it should help to show new tools that help teachers to do their work in a simpler and more rigorous way, but at the same time help to spread strategies that help the development of more meaningful and functional learning, such as those based on learning in a collaborative way. This article is intended as a basis or starting point for simple and more rigorous grouping of learners.

## Supporting information

S1 Appendix Entropy of a Stochastic Block Model (SBM) given a weighted network. More detailed information about the work by Peixoto [58].
(DOCX)

S1 Fig MECG partitions have lower entropy. Analogously as in Fig 3, we compute the fitness value based on Moreno et al. [39] in several synthetic classrooms using our MECG approach, a genetic algorithm that minimizes this fitness value, and a random grouping.
(PDF)
