# Supplementary material for: Minimum entropy collaborative groupings: A tool for an automatic heterogeneous learning group formation
Source: PLoS One. 2023 Mar 15;18(3):e0280604. doi: 10.1371/journal.pone.0280604 (PMC10016679; doi:10.1371/journal.pone.0280604)
Supplement: S1 Appendix — More detailed information about the work by Peixoto [58]. (DOCX) [file pone.0280604.s001.docx]

**Appendix**

To compute the entropy of a specific Stochastic Block Model (SBM) given a weighted network, we use the approach by [1]. We applied his python module called graph-tool to compute the entropy of a weighted SBM. In this section we will introduce how the entropy of a weighted SBM can be computed with Bayesian probability. The complete explanation can be found in Peixoto’s article and the graph-tool webpage.

One specific SBM is defined with two characteristics: (i) the partition of nodes into groups; (ii) the probability of connections between those groups. Given this information several different networks can be randomly generated. For this reason, SBMs are called a family of generative models. We are interested in the SBM that has the highest probability of generating the network that we wish to observe.

Bayesian probability computes the probability of an observation (in our case the adjacency matrix A of a network)

given some prior knowledge, the model parameters *θ* (in our case the SBM). This probability is called the likelihood, and is usually written as P(*A*|*θ*).

Since entropy measures uncertainty, it is reasonable to compute it with Bayesian probability. Graph-tool software computes the entropy S of an SBM with

$S=\ln P\left( A | \theta\right)-\ln P(\theta)$

Including the description length ln=P(*θ*) as a penalty to avoid overfitting. P(*A|θ*) clearly depends on the model parameters used. For a SBM *θ* = *{e, b}, b* is the partition of nodes into blocks, and *e* the set of edge counts between blocks. Note that these are the two characteristics for defining an SBM. If a weighted SBM is needed, we substitute *e* with the parameter *γ*, representing the sample of the weights between blocks.

**References**

1. Peixoto, T.P., 2018. Reconstructing networks with unknown and heterogeneous errors. Phys. Rev. X 8, 041011. URL: [https://link.aps.org/](https://link.aps.org/doi/10.1103/PhysRevX.8.041011) [doi/10.1103/PhysRevX.8.041011](https://link.aps.org/doi/10.1103/PhysRevX.8.041011)
