# Supplementary figures and images for: Minimum entropy collaborative groupings: A tool for an automatic heterogeneous learning group formation
Source: PLoS One. 2023 Mar 15;18(3):e0280604. doi: 10.1371/journal.pone.0280604 (PMC10016679; doi:10.1371/journal.pone.0280604)

## CHAEA

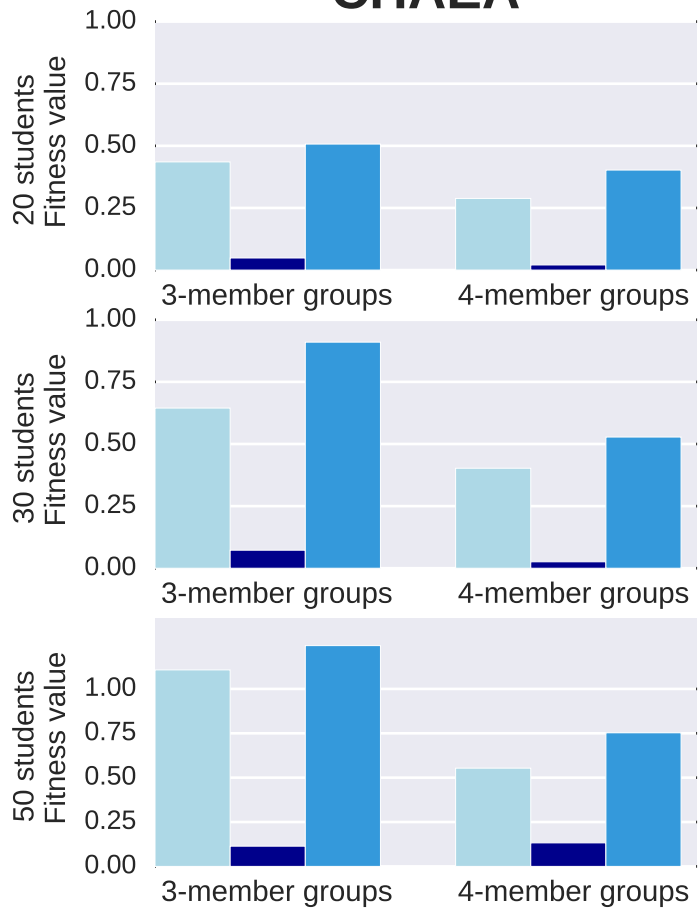

## LML

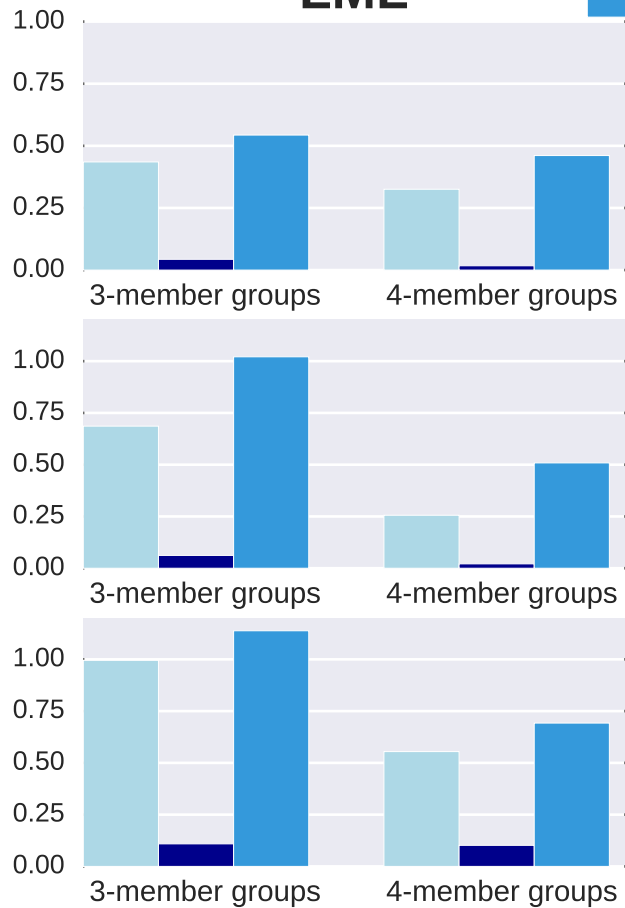

Random  
GA  
MECG

Supplement: S1 Fig — Analogously as in Fig 3, we compute the fitness value based on Moreno et al. [39] in several synthetic classrooms using our MECG approach, a genetic algorithm that minimizes this fitness value, and a random grouping. (PDF) [file pone.0280604.s002.pdf]
